# First-Principles Study of Structural, Electronic and Magnetic Properties of Metal-Centered Tetrahexahedral V_15_^+^ Cluster

**DOI:** 10.3390/nano7070164

**Published:** 2017-06-30

**Authors:** Xiaojun Li, Hongjiang Ren, Xinwei Huang, Shuna Li

**Affiliations:** The Key Laboratory for Surface Engineering and Remanufacturing in Shaanxi Province, School of Chemical Engineering, Xi’an University, Xi’an 710065, China; hjren@xawl.edu.cn (H.R.); xinweihuang@163.com (X.H.); lishuna101@163.com (S.L.)

**Keywords:** vanadium clusters, electronic structures, chemical bondings, magnetic moments

## Abstract

The V-centered bicapped hexagonal antiprism structure (**A**), as the most stable geometry of the cationic V_15_^+^ cluster, is determined by using infrared multiple photo dissociation (IR-MPD) in combination with density functional theory computations. It is found that the **A** structure can be stabilized by 18 delocalized 3c-2e σ-bonds on outer V_3_ triangles of the bicapped hexagonal antiprism surface and 12 delocalized 4c-2e σ-bonds on inner trigonal pyramidal V_4_ moiety, and the features are related to the strong *p*-*d* hybridization of the cluster. The total magnetic moments on the cluster are predicted to be 2.0 *µ*_B_, which come mainly from the central vanadium atom.

## 1. Introduction

Transition metal clusters have attracted a lot of attention because of their especially important roles in nanostructured material science [1,2,3,4,5,6,7,8,9], which are quite different from those of individual atoms or bulk phase [10,11], and present a strong size dependence on their interesting physicochemical properties, e.g., optical [12,13,14], magnetic [15,16] and catalytic [17,18,19] properties, serving in various industrial application [20]. In particular, vanadium clusters are of special interest, due to their magnetic properties [21,22,23,24] and novel catalysis [25,26], which are closely related to their electronic structures. It is well-known that one of the major challenges for understanding the fascinating electronic properties is to determine the cluster structure, which can be solved well by using a combination of state-of-the-art experimental and theoretical studies. For example, Fielicke et al. [27,28] explored the geometric structures of vanadium clusters using infrared multiple photon dissociation (IR-MPD) spectroscopy and density functional theory (DFT) computations, and identified the ground-state structures of some vanadium clusters. Also, the stable structures of large vanadium clusters have been theoretically determined by classical potentials, such as tight-binding potential [23,29]. In 2001, Taneda et al. [30] performed molecular-dynamics (MD) simulations on the disordered structures of the V*_n_* clusters (*n* = 2–17, 55, 147 and 309), and found that the globally stable structures of small clusters are in agreement with first-principle calculations. Meanwhile, vanadium clusters have been experimentally measured using several experimental techniques, e.g., using the IR-MPD for V*_n_*^+^ (*n* = 6–23) [31], photoelectron spectroscopy (PES) for V*_n_*^−^ (*n* = 3–65) [16,32], and collision-induced dissociation (CID) of V*_n_*^+^ (*n* = 2–20) with Xe by guided ion beam mass spectrometry [33]. 

**Figure 1:**
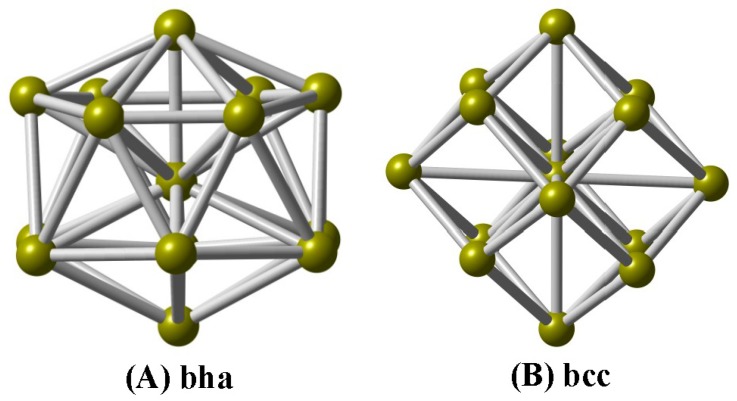
Two low-lying structures of the cationic V_15_^+^ cluster. (**A**) bicapped hexagonal antiprism (*bha*); (**B**) body-centered cube (*bcc*).

In this paper, we explore the geometric structures of V-centered bicapped hexagonal antiprisms (**A** *bha*, Figure 1) and body-centered cubes (**B** *bcc*) for the cationic V_15_^+^ cluster using a combination of DFT computations and IR-MPD spectroscopy, and determine the ground-state structure to be a V-centered tetrahexahedral geometry (**A**), which reproduces experimentally observed spectra well. Based on the assigned structure, the electronic structures, chemical bondings, and magnetic properties are investigated. 

## 2. Materials and Methods

Cluster structure computations were performed on the Gaussian 09 suite of programs [34] using the following four density functionals: (a) PBE [35,36]; (b) BLYP [37,38]; (c) BPW91 [37,39]; and (d) BP86 [37,40,41]. The Karlsruhe split-valence (def-SVP) basis set, augmented with polarization functions and the all-electron 6-311+G(d) basis set, was applied for the two vanadium clusters. In the computation, the singlet and triplet spin electronic states (spin multiplicities 2*S* + 1 = 1, 3) were taken into account for the energy evaluation of the low-lying isomers. The two low-lying structures were optimized without any symmetry constraints. Harmonic vibrational frequencies were employed to confirm that the two structures correspond to real local minima, and zero-point vibrational corrections were included in the relative energies. 

To aid the comparison with the experimental IR-MPD spectrum, the calculated infrared stick spectra were convoluted with a Gaussian function with a full width at half-maximum (FWHM) of 6 cm^−1^. In order to fit well with the experimental data, a frequency scaling factor of 0.87 was applied, similar to previous studies [28,31]. The density-of-states (DOS) spectra were convoluted utilizing the GaussSum 2.2 program [42] with a full-width at half maximum (FWHM) of 0.3 eV. The molecular orbitals were plotted with the isodensity surfaces (0.02 e^1/2^/(Bohr)^3/2^), and the molecular graphs were visualized using the VMD program [43]. Chemical bonding analyses were performed using the adaptive natural density partitioning (AdNDP) method proposed by Zubarev and Boldyrev [44]. 

## 3. Results and Discussion

### 3.1. Structural Determination

Structurally, we can see that the **A** isomer of the V_15_^+^ cluster is a V-centered tetrahexahedral structure (Figure 1A), consisting of a bicapped hexagonal antiprism (*bha*), in which the hexagonal faces are capped by a V atom above and below. Interestingly, the **A** isomer can be regarded as a wheel structure, and the V–V–V central axle can be surrounded by the V_12_ hexagonal antiprism. The **A** structure is found to be consistent with Fe@Si_14_ [45] and Fe@Ge_14_ [46] clusters. At the PBE/def-SVP level of theory, the average V–V bond lengths for the **A** isomer are predicted to be 2.506 Å, while a large average coordination number (6.67) is obtained on the vanadium atoms, which are close to previous calculations for V_15_ [23], i.e., 2.503 Å and 6.67 for V–V bond lengths and coordination number, respectively. The **B** isomer is a stuffed fullerene-like structure (Figure 1B), consisting of a body-centered cube (*bcc*, 8 + 1 atoms) framework with six tetrahedral pyramids on each face. One can find that the average V–V bond lengths for the **B** isomer are deemed to be 2.511 Å, while the average coordination number is also 6.67; there are no other experimental or theoretical data for comparison. The same structure was predicted for the isovalent Ta_15_^+^ cluster [6]. 

**Figure 2:**
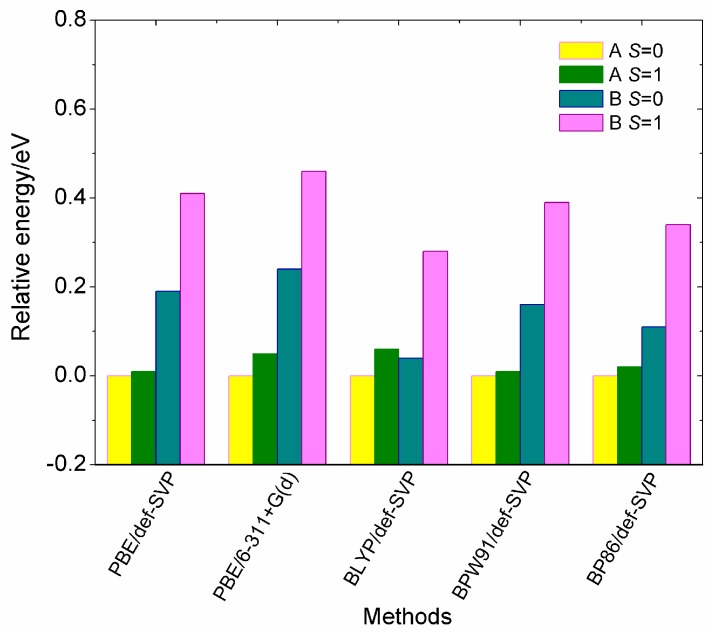
Comparison of relative energies of the two low-lying isomers (**A** and **B**) for the cationic V_15_^+^ cluster in different spin states, obtained by using the five different methods. All the calculations are based on the zero-point corrected total energies upon full optimizations.

Figure 2 compares the relative energies of two low-lying isomers (**A** and **B**) for the V_15_^+^ cluster in singlet (*S* = 0) and triplet (*S* = 1) spin states, which are calculated from the five different theoretical methods. The relative energy can be evaluated by the energy differences of the two low-lying isomers in different spin states relative to the lowest-energy states (**A**, *S* = 0). According to the calculated results, it is found that the PBE, BPW91 and BP86 functionals provide similar energy orderings, whereas the BLYP functional predicts a **B** isomer with *S* = 0 to be more stable than **A** with *S* = 1 by 0.02 eV. At the same time, it clearly shows that the **A** geometry with two spin states is more stable than the **B** geometry, which is different from the results reported in [28], which predicted that the **B** structure is more stable in energy than the **A** geometry. Moreover, it is seen from Figure 2 that the **A** isomer with spin singlet (*S* = 0) is energetically preferred, and its spin triplet (*S* = 1) is less stable than the singlet state, having a higher energy by only 0.01–0.06 eV. Conversely, the **B** structure has larger relative energies by up to 0.46 eV among these theoretical methods. In addition, we have also performed global optimizations for the cluster using the semi-empirical Gupta potential with the basin-hopping method, and found that the **A** structure is more stable than the **B** structure by 0.39 eV. Thus, one can see from Figure 2 that the two spin states of the **A** isomer display a very small energy difference, but we cannot predict with certainty which of two spin states will be the true ground-state.

**Figure 3:**
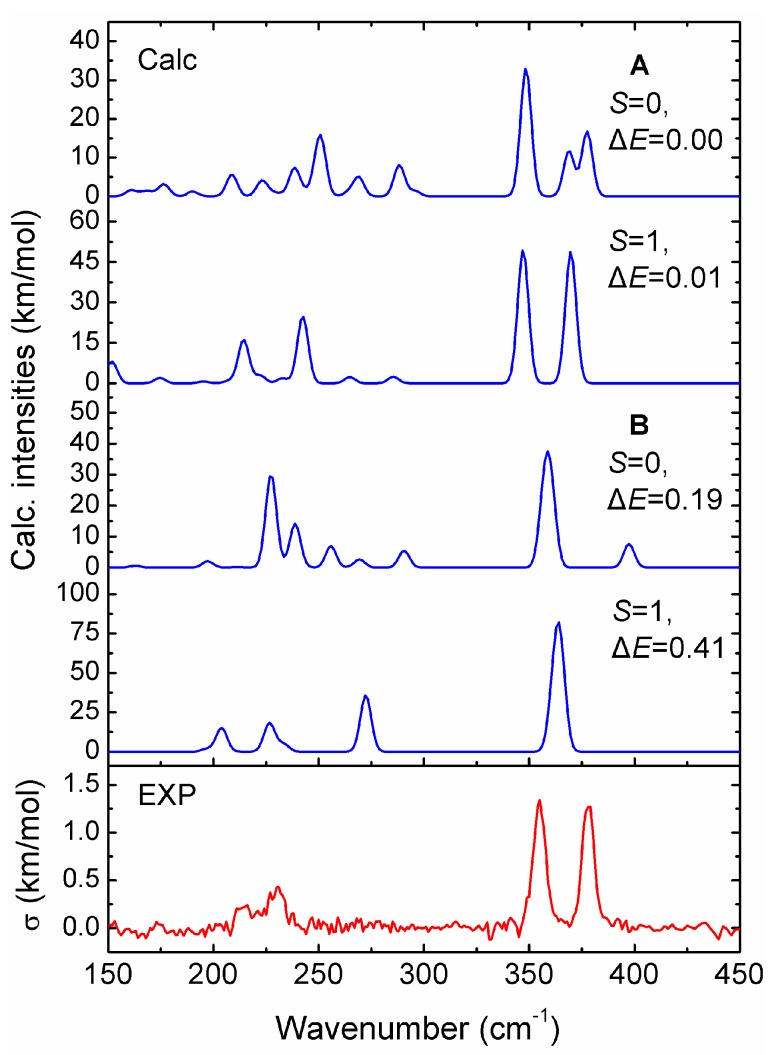
Comparison of experimental IR-MPD spectrum (lower panel) of V_15_^+^-Ar cluster with the calculated IR spectra (upper panels) of the two low-lying structures (**A** and **B**) in different spin states (*S* = 0 and 1), obtained from the PBE/def-SVP level of theory. The σ symbol represents the experimental IR cross section. A Gaussian band with the full width at half-maximum (FWHM) of 6 cm^−1^ was used. A scaling factor of 0.87 was applied to correct all the calculated vibrational frequencies.

In order to test the reliability of the prediction for the V_15_^+^ cluster based on the stable structures, we compare the calculated infrared spectra of the two low-lying isomers (**A** and **B**) in different spin states obtained at the PBE/def-SVP level of theory, with the experimental IR-MPD spectrum from previous work [31], to correctly obtain structural assignments for the cluster. As shown in Figure 3, the experimental IR-MPD spectrum of V_15_^+^-Ar shows four characteristic absorptions with two intense bands at around 355 and 378 cm^−1^, and two weak bands at around 214 and 232 cm^−1^. It is apparent that the experimental spectroscopic bands are in excellent agreement with the theoretical prediction for the low-lying **A** isomer with spin triplet (*S* = 1). By comparison, the former two main experimental bands correspond to calculated bands centered on 347 and 370 cm^−1^, respectively, in which the first intense peak is primarily caused by the V–V stretching vibrations along the central V–V–V axis, while another intense peak is largely attributed to the horizontal wagging vibrations on the central V atom of the V–V–V axis. Other weak experimental bands at 214 and 232 cm^−1^ are reproduced well by those predicted at 214 and 242 cm^−1^, respectively. Thus, the **A** structure with spin triplet (*S* = 1) should be present in the molecular beam, and should contribute significantly to the experimental IR spectrum, which updates the previous report for the cluster [28].

### 3.2. Electronic Structures

**Figure 4:**
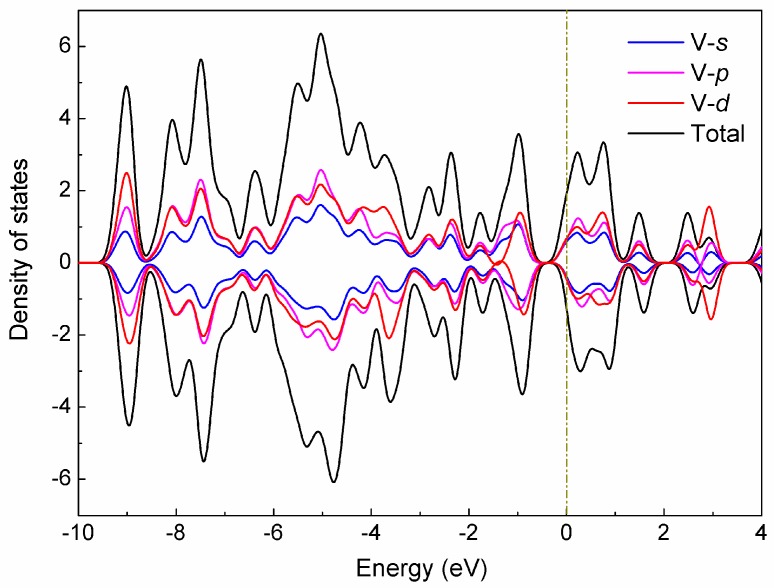
Density of states (DOS) of the low-lying **A** structure for the V_15_^+^ cluster. The orbital contributions (V-*s*, V-*p*, and V-*d*) of the cluster to the TDOS spectrum are labeled. The Fermi level is shifted to zero.

To systematically investigate the electronic structures of the V_15_^+^ cluster, we calculated the total (TDOS) and partial (PDOS) density of states for the low-lying **A** structure in terms of Mulliken population analysis [47], which includes the orbital contributions (V-*s*, V-*p*, and V-*d*) of the vanadium atom in the cluster to the TDOS, as depicted in Figure 4. As evidenced by the diagram, a strong hybridization occurred between the *s*, *p*, and *d* states, especially for *p*-*d* hybridization, and these hybridizations are mainly responsible for the structural stabilization of the cluster. It can also be observed from Figure 4 that the electronic features largely originate from the *d* states near the Fermi level, while the *s* and *p* states produce nearly non-negligible contributions. Meanwhile, the up-spin and down-spin bands of these different states are not substantially split for the **A** structure with spin triplet (*S* = 1), which may result in a smaller magnetic moment.

To further understand the electronic properties of the low-lying **A** structure, we also explore the up-spin orbital contributions in the DOS spectra (see Figure S1), involving the highest occupied molecular orbital (HOMO) and the lowest unoccupied molecular orbital (LUMO). We can clearly see that there are three strong DOS peaks, located at −9.02, −7.49, and −5.03 eV, respectively. More impressively, the HOMO-32 orbital at −9.02 eV has a *p*-type MO pattern, which is contributed to significantly by the *s*-*p* hybridization between the interior V atom and surrounding V atoms, with the 26.44% V-4*s* and 32.16% V-4*p_y_* states. The strongest occupied orbital peak (HOMO-12), located at −7.49 eV, is mainly attributable to the shell contributions (24.33% V-3*d_xy_*, 21.05% V-3*d_z_*^2^ states) of the completely delocalized σ-type MO on the hexagonal antiprism V atoms, with small components from 14.79% V-4*p_z_* state of the vanadium atoms. Obviously, the HOMO and LUMO orbitals have weak DOS peaks, where the HOMO orbital originates largely from the shell contributions of 20.24% V-3*d_xy_* and 31.93% V-3*d_x_*^2^*_y_*^2^ states, while the LUMO significantly involves the 24.77% V-4*s* and 35.34% V-3*d_z_*^2^ states, which results in the formation of strong localized atomic orbitals on each vanadium atom, especially for two capped V atoms (Figure S1). One can see that the HOMO-LUMO gap for the **A** isomer is predicted to be 0.38 eV, and the energy gap is mostly associated with the electron distributions of the HOMO and LUMO orbitals. It is expected that these theoretical results will be helpful to understand the electronic properties of the V_15_^+^ cluster, and gain insight into the origins of its stable geometry.

### 3.3. Chemical Bonding Analyses

In order to gain insight into the chemical bonding of the V_15_^+^ cluster, the *n*-center two-electron (*n*c-2e, *n* going from one (lone-pair) to the maximum number of clusters) bonds were, for the first time, explored using the adaptive natural density partitioning (AdNDP) method proposed by Zubarev and Boldyrev [44], which has been successfully used to reveal bonding characteristics not only for organic aromatic molecules [48,49], but also for boron [50,51] and transition-metal doping clusters [52,53,54]. 

**Figure 5:**
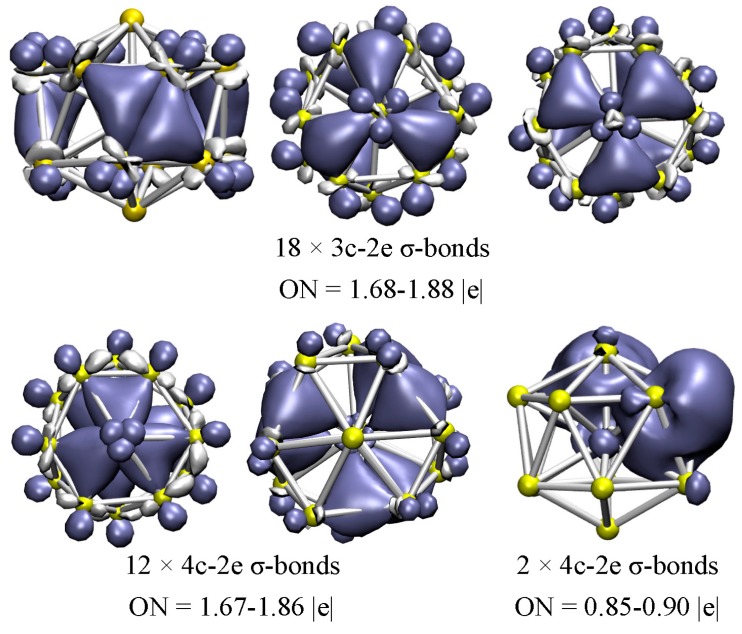
AdNDP chemical bonding analyses of the low-lying **A** structure for the V_15_^+^ cluster. ON denotes the electron occupation number and is close to the ideal population of 2.00 |e|.

As mentioned above, the low-lying **A** structure is a bicapped hexagonal antiprism (**bha**) with an endohedral V atom. According to the AdNDP results (Figure 5), a total of 18 delocalized 3c-2e σ-bonds can be readily identified: six 3c-2e σ-bonds (ON = 1.84–1.88 |e|) on the outer V_3_ triangles of the hexagonal antiprism surface, and twelve 3c-2e σ-bonds (ON = 1.68–1.81 |e|) above and under the V_3_ triangles of the bicapped hexagonal faces. On the other hand, 12 delocalized 4c-2e σ-bonds are identified, with ON = 1.67–1.86 |e|, which are located on the inner trigonal pyramidal V_4_ moiety, involving the 12-atom outer ring and endohedral V atom, while none of the valence electrons in this structure are delocalized π-bonds or localized 2c-2e bonds. Accordingly, it is interesting that the low-lying **A** structure can be stabilized by the 3c-2e σ-bonds on the outer shell, and the 4c-2e σ-bonds on the inner shell. The two extra electrons in the open-shell **A** structure are clearly delocalized over the two V_4_ trigonal pyramid, and the ONs of the two σ-bonds are 0.85–0.90 |e|. In addition, the remaining twelve valence electrons can be characterized as six totally delocalized molecular orbitals (MOs) with ON = 2.00 |e|, distributed over the cage surface of the structure (see Figure S2).

### 3.4. Magnetic Moments and Charge Transfers

Local magnetic moments and charge transfers were performed to understand the magnetic properties of the cationic V_15_^+^ cluster, see Table S1. One can see that the average magnetic moments for the low-lying **A** and **B** structures in spin triplet (*S* = 1) are predicted to be 0.13 *µ*_B_ per atom, and are primarily produced by the central vanadium atom, e.g., 0.27 and 0.22 *µ*_B_ for **A** and **B**, respectively. Meanwhile, the average local moments for the first shell atoms are calculated to be 0.18 and 0.16 *µ*_B_ for **A** and **B**, respectively, obtained by using the PBE/def-SVP level of theory. The charge transfer can be used to reflect the interaction between the central vanadium atom and the cluster surface. Obviously, the charge largely transfers the 0.95 electron from the cluster surface to the central vanadium atom, which governs the structural stability of the endohedral cluster. It is worth mentioning that the strong interactions between the central vanadium atom and cluster surface are consistent with the chemical bonding analysis as discussed above, i.e., 12 delocalizedly inner 4c-2e σ-bonds. The electronic dipole moments are closely related to the structural features and electronic properties of the clusters. At the PBE/def-SVP level of theory, the theoretical dipole moments of the low-lying **A** structure in spin singlet and triplet are predicted to be 4.26 and 4.62 D, respectively, with the cluster’s symmetries being largely responsible for the moderate dipole moments.

## 4. Conclusions

In summary, the geometric structures of V-centered bicapped hexagonal antiprisms (**A**) and body-centered cubes (**B**) for cationic V_15_^+^ cluster are identified by using infrared multiple photo dissociation in combination with density functional theory computations. It is found that the **A** isomer with spin triplet reproduces well the experimentally observed spectrum, and the characteristic peaks are properly assigned according to theory. Based on the AdNDP analysis, we can see that the **A** isomer can be stabilized by 18 delocalized 3c-2e σ-bonds on the outer V_3_ triangles of the bicapped hexagonal antiprism surface, and 12 delocalized 4c-2e σ-bonds on the inner trigonal pyramidal V_4_ moiety. Meanwhile, there is strong hybridization between the *s*, *p*, and *d* states, especially for *p*-*d* hybridization, which could further explain the electronic structures of the cluster, with the local magnetic moments of the cluster originating mainly from the central vanadium atom. Thereby, our results will inevitably stimulate future theoretical and experimental studies for the exploration of novel vanadium-based catalytic materials.

## Supplementary Materials

The following are available online at http://www.mdpi.com/2079-4991/7/7/164/s1. Figure S1: Up-spin DOS and MOs of the low-lying **A** structure for V_15_^+^, Figure S2: Six totally delocalized bonding patterns for V_15_^+^ revealed by AdNDP analysis, Table S1: Magnetic moments of two low-lying isomers (**A** and **B**) for V_15_^+^, Table S2: Optimized atomic coordinates of the lowest-energy **A** isomer for V_15_^+^.
