# Supplementary material for: First-Principles Study of Structural, Electronic and Magnetic Properties of Metal-Centered Tetrahexahedral V15+ Cluster
Source: Nanomaterials (Basel). 2017 Jun 30;7(7):164. doi: 10.3390/nano7070164 (PMC5535230; doi:10.3390/nano7070164)
Supplement: Supplementary file 1 [file nanomaterials-07-00164-s001.pdf]

Supplementary Material

First-Principles Study of Structural, Electronic and Magnetic Properties of Metal-Centered Tetrahexahedral V_{15}^{+} Cluster

Xiaojun Li, Hongjiang Ren, Xinwei Huang, Shuna Li

The Key Laboratory for Surface Engineering and Remanufacturing in Shaanxi Province,

School of Chemical Engineering, Xi'an University, Xi'an 710065, Shaanxi, P. R. China

Contents:

- Up-spin DOS and MOs of the low-lying **A** structure for V_{15}^{+}, Fig. S1
- Six totally delocalized bonding patterns for V_{15}^{+} revealed by AdNDP analysis, Fig. S2
- Magnetic moments of two low-lying isomers (**A bha** and **B bcc**) for V_{15}^{+}, Table S1
- Optimized atomic coordinates of the lowest-energy A isomer for V_{15}^{+}, Table S2

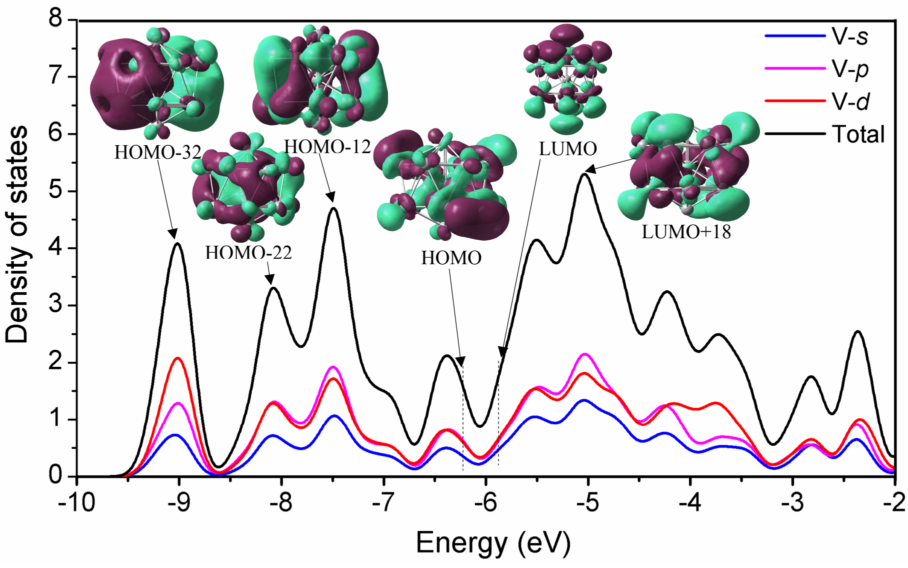

Fig. S1 Up-spin density of states (DOS) of the low-lying **A** structure for V_{15}^{+}, obtained at the PBE/def-SVP level of theory. The molecular orbitals are related to the mainly up-spin DOS bands.

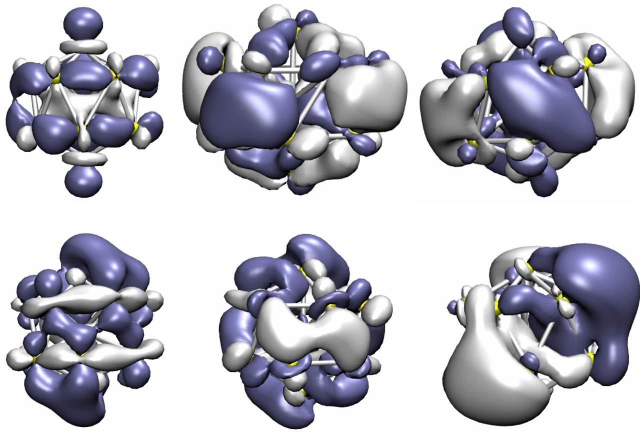

six totally delocalized MOs
ON = 2.00 |e|

Fig. S2 Six totally delocalized bonding patterns (molecular orbitals) for the V_{15}^{+} cluster, revealed by AdNDP method[1] at the PBE/def-SVP level of theory, ON denotes the electron occupation number. All of the molecular isovalue graphs were visualized using the VMD program.[2]

1. D. Y. Zubarev and A. I. Boldyrev, *Phys. Chem. Chem. Phys.*, 2008, **10**, 5207–5217.
2. W. Humphrey, A. Dalke and K. Schulten, *J. Mol. Graphics* 1996, **14**, 33–38.

Table S1 Magnetic moments (in μ_B) of two isomers A (bicapped hexagonal antiprism, **bha**) and B (body-centered cube, **bcc**) for the V_{15}^+ cluster: μ is the average magnetic moment per atom; μ_1, μ_2, and μ_3 are the average local magnetic moments for center, first shell, and second shell atoms, respectively.

Isomers	Spin states	Magnetic moments[a]			
		μ	μ_1	μ_2	μ_3
**A bha**	S=1	0.13	+0.27	+0.18	−0.20
**B bcc**	S=1	0.13	+0.22	+0.16	+0.09

[a]The magnetic moments were calculated by using the PBE/def-SVP level of theory.

Shell definations:

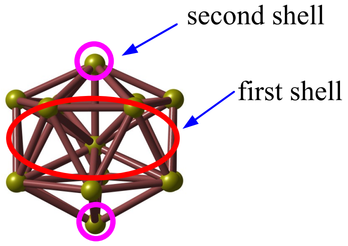

A hp

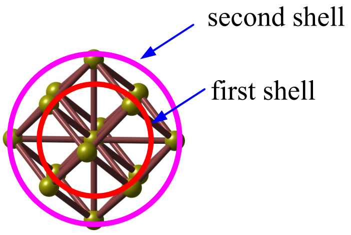

B bcc

Table S2 Optimized atomic coordinates of the lowest-energy isomer (A) in different spin states, for the cationic V_{15}^{+} cluster.

isomers	Atom	X(Å)	Y(Å)	Z(Å)	isomers	Atom	X(Å)	Y(Å)	Z(Å)
A	V	3.3691151	-0.624274	0.3435209	A	V	3.3050438	-0.745791	0.3772514
S=0	V	0.8033133	-0.571607	0.3228301	*S*=1	V	0.7648149	-0.514081	0.2876804
	V	-1.398184	0.7329318	0.8625263		V	-1.423715	0.7852288	0.8638335
	V	2.1499128	1.3849843	-0.738424		V	2.3249223	1.339609	-0.677379
	V	0.4927554	0.2382071	2.6761637		V	0.2880789	0.2887488	2.6640119
	V	0.1930817	-1.915413	-1.844635		V	0.1026531	-1.897812	-1.823589
	V	2.0179956	-0.794024	-1.988596		V	1.8887534	-0.707167	-1.997021
	V	-0.289177	-2.874372	0.6320758		V	0.0676414	-2.964207	0.4785844
	V	1.975505	-2.587636	-0.472949		V	2.0037323	-2.695484	-0.424587
	V	1.6142764	-1.66006	2.100838		V	1.6550588	-1.736648	2.0173355
	V	0.6776367	2.0110845	0.6996041		V	0.6946023	2.0247012	0.5541353
	V	2.6504676	0.7680785	1.6595005		V	2.5365507	0.7406361	1.7338646
	V	-1.056835	-1.510841	1.9493188		V	-0.963837	-1.356174	2.0496897
	V	-0.091035	0.6532018	-1.455887		V	-0.128742	0.7092895	-1.441838
	V	-1.656214	-0.95719	-0.441095		V	-1.662943	-0.977777	-0.357177
